# Assessment of *MGMT* methylation status using high-performance liquid chromatography in newly diagnosed glioblastoma

**DOI:** 10.1186/s13148-020-00968-5

**Published:** 2020-11-17

**Authors:** Mitsuto Hanihara, Kunio Miyake, Atsushi Watanabe, Yuriko Yamada, Naoki Oishi, Tomoyuki Kawataki, Takeshi Inukai, Tetsuo Kondo, Hiroyuki Kinouchi

**Affiliations:** 1 Departments of Neurosurgery, University of Yamanashi, Chuo, Yamanashi Japan; 2 Department of Health Sciences, Interdisciplinary Graduate School of Medicine and Engineering, University of Yamanashi, 1110 Shimokato, Chuo, Yamanashi 409-3898 Japan; 3 Department of Pediatrics, University of Yamanashi, Chuo, Yamanashi Japan; 4 Tsukuba Research Institute, Research and Development Division, Sekisui Medical Co., Ltd., Ryugasaki, Japan; 5 Department of Pathology, Interdisciplinary Graduate School of Medicine and Engineering, University of Yamanashi, Chuo, Yamanashi Japan

**Keywords:** *MGMT*, DNA methylation, Glioblastoma, HPLC, Prognostic factor

## Abstract

**Background:**

The utility of O^6^-methylguanine-DNA methyltransferase (MGMT) gene promoter methylation status as a prognostic marker in patients with glioblastoma (GBM) has been established. However, the number of CpG sites that must be methylated to cause transcriptional silencing remains unclear, and no significant consensus exists on the optimal method of assessing *MGMT* methylation. We developed a new high-performance liquid chromatography (HPLC) method that enables accurate analysis of DNA methylation levels using long PCR products. In the present study, we analyzed the *MGMT* methylation status of 28 isocitrate dehydrogenase–wild-type GBMs treated with temozolomide using ion-exchange HPLC and set the optimal cutoff values.

**Results:**

We designed three primers for separate regions (regions 1–3) that had 21 to 38 CpGs for PCR and validated the *MGMT* promoter methylation status using frozen samples. There was a strong correlation between HPLC and bisulfite sequencing results (*R* = 0.794). The optimal cutoff values for *MGMT* methylation in HPLC were determined to allow differentiation of patient prognosis by receiver operating characteristic curve analysis. The cutoff values were 34.15% for region 1, 8.84% for region 2, and 36.72% for region 3. Kaplan–Meyer curve analysis estimated that the most differentiated prognosis was enabled in the setting of 8.84% methylation of *MGMT* in region 2. Progression-free survival and overall survival were significantly longer for patients in this setting of region 2 methylation (*p* = 0.00365 and *p* = 0.00258, respectively).

**Conclusions:**

The combination of our HPLC method and the original primer setting provides a new standard method for determination of *MGMT* methylation status in patients with GBM and is useful for refining *MGMT*-based drug selection.

## Background

Glioblastoma (GBM) is the most common brain tumor in adults; it has an aggressive lethal nature, with a median survival of only 12–15 months, despite the standard established therapy of maximum resection followed by radiation and chemotherapy [1–3]. GBM chemotherapy is based on drugs that alkylate the DNA in the O^6^-position of guanine, such as temozolomide (TMZ). O^6^-methylguanine-DNA methyltransferase (MGMT) is a protein that repairs the naturally occurring mutagenic DNA lesion O^6^-methylguanine back to guanine and prevents mismatch and errors during DNA replication and transcription. Since epigenetic silencing of the *MGMT* gene via promoter methylation is associated with loss of *MGMT* expression [4, 5], analysis of *MGMT* methylation status has become a key prognostic marker of TMZ.

In GBM patients, the relevance of *MGMT* promoter methylation as a predictive marker for TMZ effectiveness has been strengthened by a randomized trial [6]. Furthermore, it has been reported that *MGMT* methylation is significantly correlated with prognosis of glioma patients treated with TMZ, with or without adjustment of isocitrate dehydrogenase (IDH) 1/2 status [7, 8]. Based on these results, *MGMT* methylation analysis has been intensely investigated in several clinical trials [6, 8, 9].

Despite considerable research, consensus regarding the optimal method for *MGMT* gene promoter methylation assessment and the optimal promoter region for *MGMT* methylation analysis is lacking. The CpG island of *MGMT* exhibits heterogeneous methylation patterns, and it is therefore possible to fail to detect methylation when only analyzing a specific area [10]. Genome sequencing analysis can overcome this problem; however, it is costly. Previous studies have identified two regions (the promoter and gene body) that are significantly correlated with *MGMT* expression [11]. However, due to the difficulty of designing a primer for the GC-rich promoter area and the lack of a method that enables analysis of long PCR products at low cost, the region of the gene body is generally selected as the target for *MGMT* methylation analysis [12]. To demonstrate the effectiveness of methylation analysis in the promoter region, a new method for analyzing longer PCR products is required.

We developed an anion-exchange, high-performance liquid chromatography (HPLC) column for the detection of methylated DNA, thus enabling the analysis of long PCR products. HPLC provides rapid and accurate quantification of DNA methylation levels within 10 min without the need for time-consuming pretreatment of the PCR products [13]. In the present study, we identified three original primers and determined the *MGMT* promoter methylation status in newly diagnosed GBM patients using this HPLC method and set the optimal cutoff values.

## Results

**Fig. 1:**
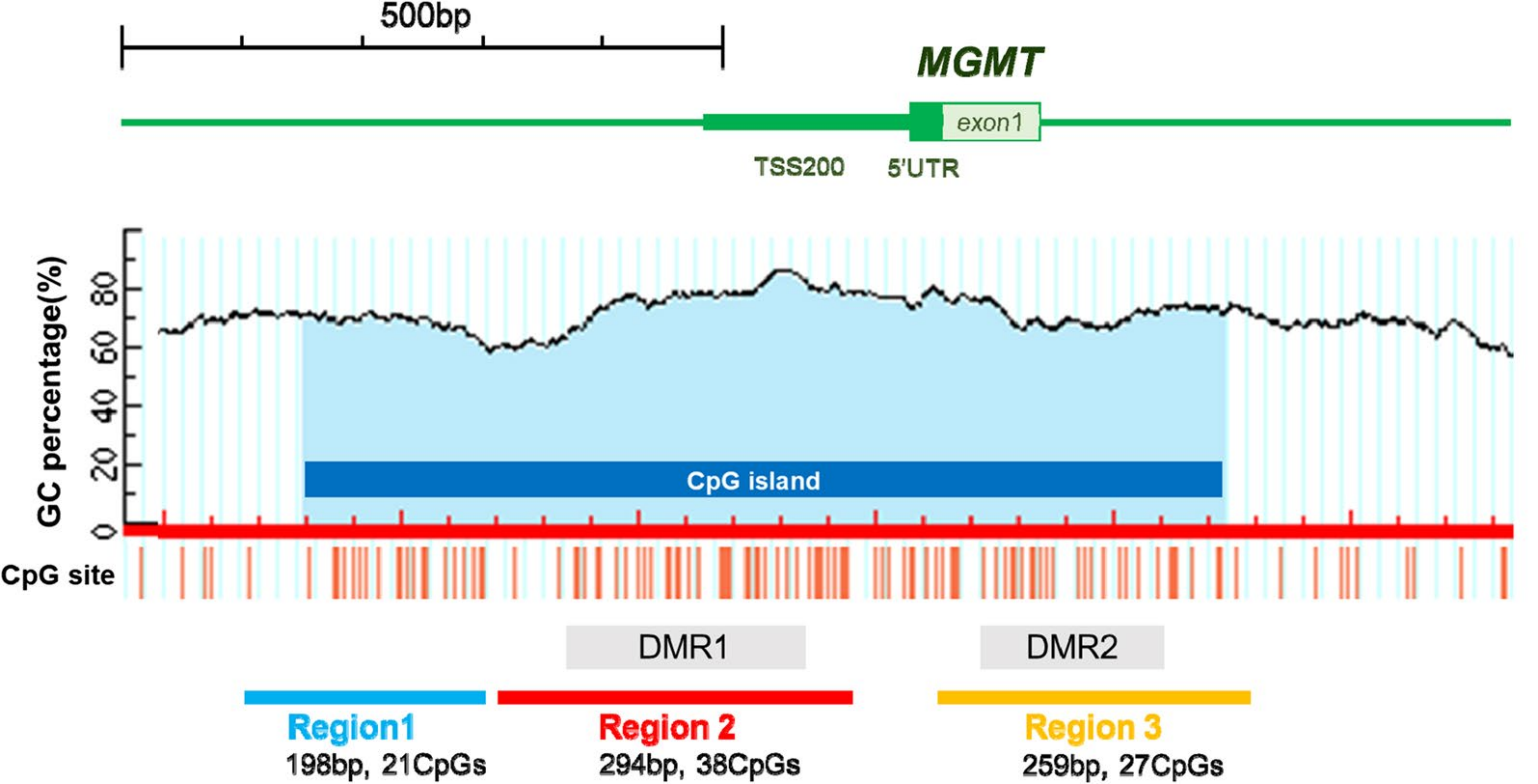
Schematic representation of the *MGMT* promoter analyzed in the present study

We identified three separate primer regions: region 1 (198 bp, 21 CpGs), region 2 (294 bp, 38 CpGs), and region 3 (259 bp, 27 CpGs) (Fig. 1, Table 1). Previous studies identified two distinct regions of *MGMT* CpG islands, known as differentially methylated region (DMR) 1 and 2, which were demonstrated to correlate with transcriptional silencing [10, 14, 15]. Our region 2 encompassed DMR 1, whereas region 3 encompassed DMR 2 (Fig. 1).

**Table 1:** List of bisulfite PCR primers

	Primer sequence (5′–3′)	Amplicon size (bp)
Region 1	F: GGTAAATTAAGGTATAGAGTTTTAGG	198
	R: AAAACCTAAAAAAAACAAAAAAAC	
Region 2	F: GGTTTGGGGGTTTTTGATTAG	294
	R: CCTTTTCCTATCACAAAAATAATCC	
Region 3	F: GGATATGTTGGGATAGTT	259
	R: ACAACACCTAAAAAACACTTAAAAC	

**Fig. 2:**
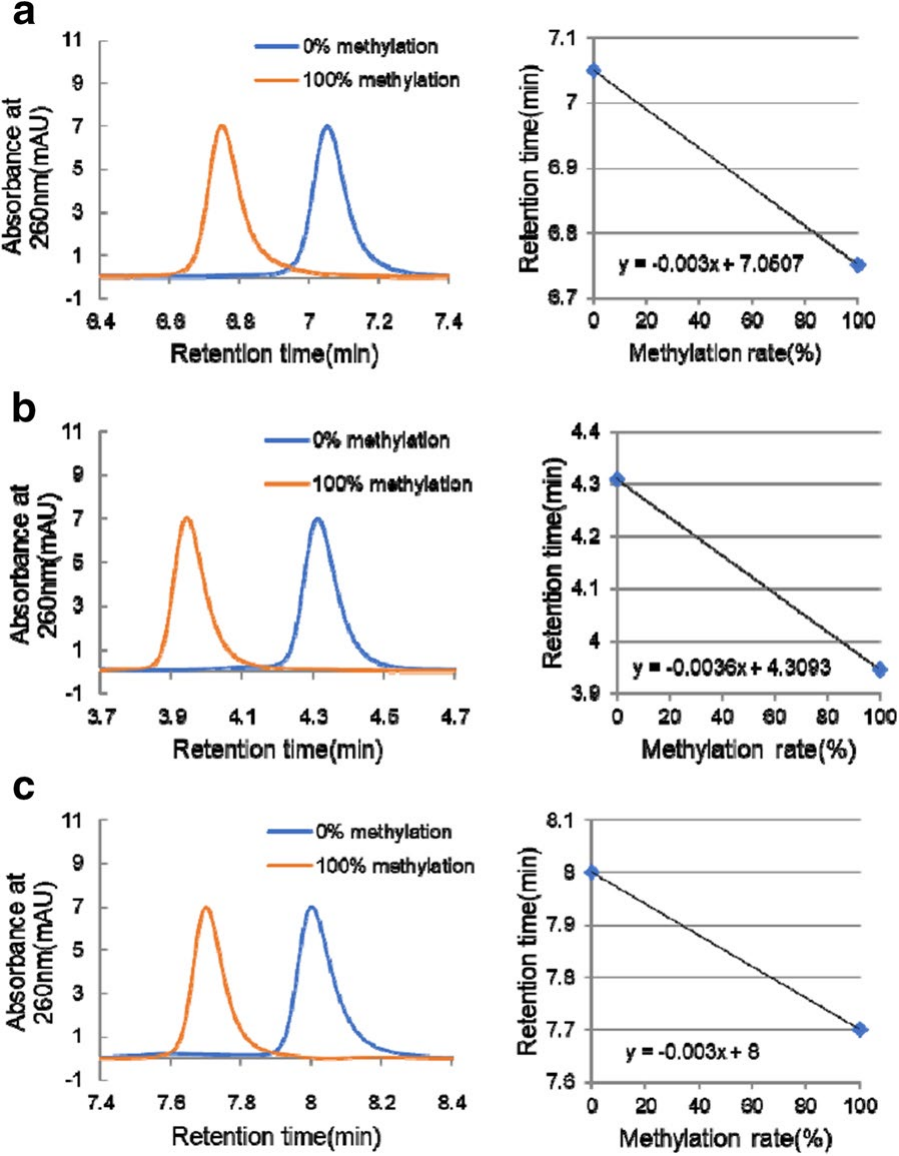
DNA methylation analysis by HPLC. Chromatograms of synthetic DNA fragments corresponding to 0% (TPG) and 100% (CpG) methylation (**a**–**c**)

The chromatograms of three regions are shown in Fig. 2. The peaks derived from synthetic DNA fragments corresponding to 0% and 100% methylation were completely separated and detected in region 1 between 6.5 and 7.5 min (Fig. 2a), in region 2 between 3.5 and 4.5 min (Fig. 2b), and in region 3 between 7.5 and 8.5 min (Fig. 2c).

**Fig. 3:**
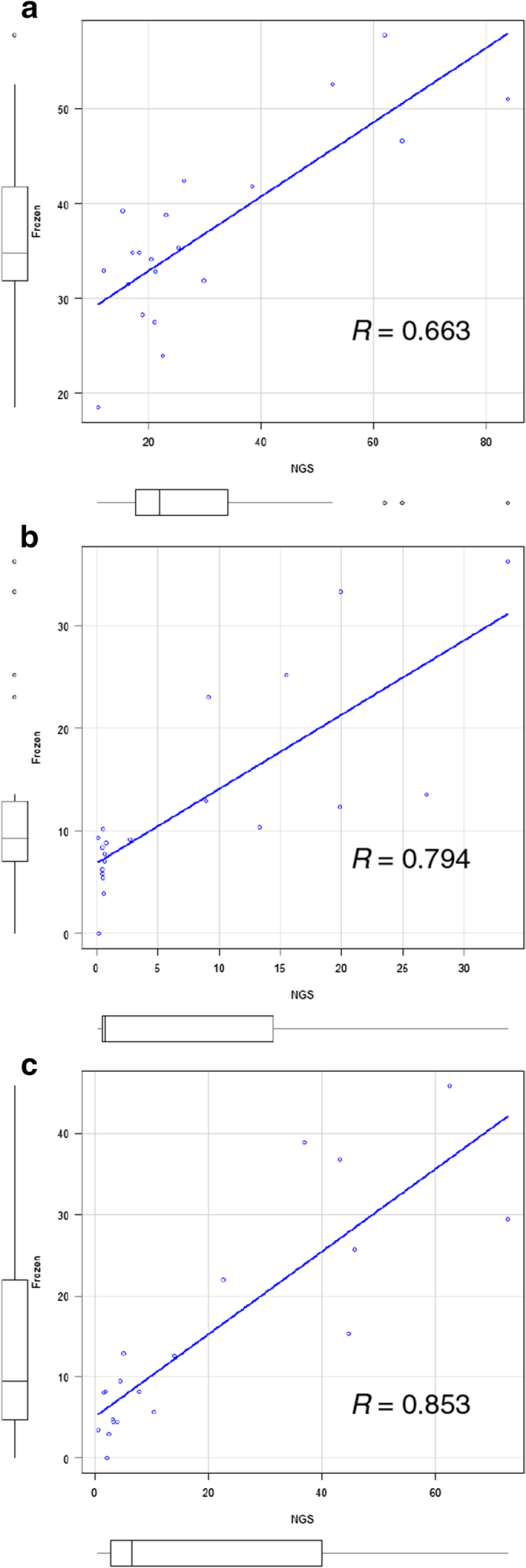
Correlation of results obtained on HPLC and NGS. Agreement between HPLC and NGS in *MGMT* methylation using Spearman’s rank correlation for region 1 (**a**), region 2 (**b**), and region 3 (**c**)

To verify the validity of the HPLC method for DNA methylation analysis, we compared the methylation status by HPLC and bisulfite sequencing using a next-generation sequencing (NGS) approach. A strong correlation in average *MGMT* methylation was observed for the frozen samples analyzed by HPLC and NGS for each primer pair (Fig. 3).

**Fig. 4:**
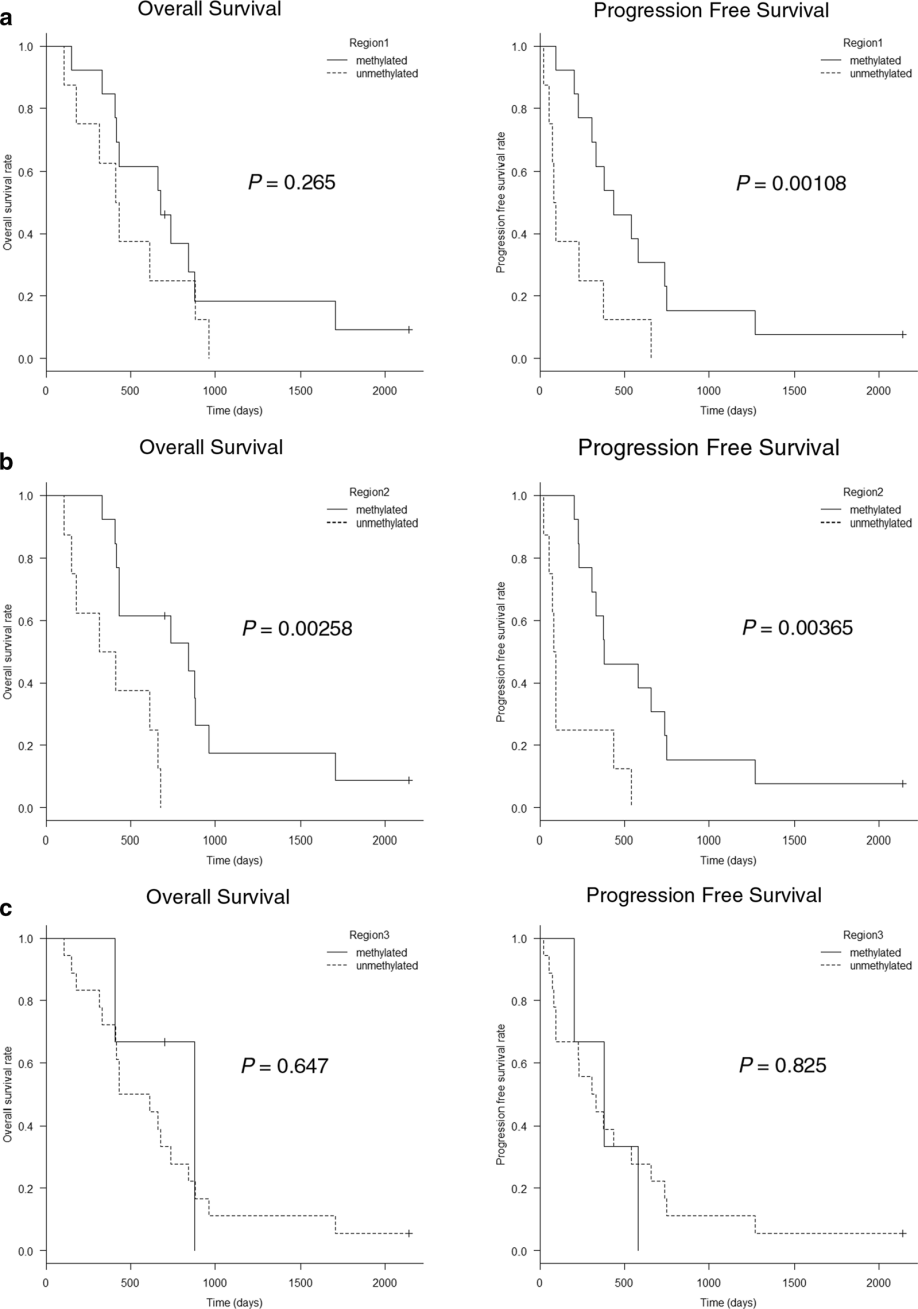
Kaplan–Meier curve based on *MGMT* promoter methylation status determined using frozen samples. For region 1, patients whose tumors were methylated exhibited significantly longer PFS than patients with unmethylated tumors (**a**). For region 2, patients whose tumors were methylated exhibited significantly longer OS and PFS than patients with unmethylated tumors (**b**). For region 3, no statistically significant differences in survival were observed between methylated and unmethylated tumors (**c**)

HPLC analysis was performed using frozen tumor samples from 21 patients, and *MGMT* methylation status could be determined for all patients. To determine the prognostic capability of the HPLC method, receiver operating characteristic (ROC) curve analysis was used to estimate the optimal cutoff values. The cutoff values for discriminating methylated and unmethylated *MGMT* were 34.15% for region 1, 8.84% for region 2, and 36.72% for region 3. Using these cutoff values, *MGMT* promoter methylation was observed in 61.9% of region 1; 61.9% of region 2, and 14.3% of region 3. Unadjusted Kaplan–Meier plots of overall survival (OS) and progression-free survival (PFS) are shown in Fig. 4. For region 1, patients whose tumors were methylated exhibited significantly longer PFS than patients with unmethylated tumors (*p* = 0.00108) (Fig. 4a). For region 2, patients whose tumors were methylated exhibited significantly longer OS (*p* = 0.00258) and PFS (*p* = 0.00365) than patients with unmethylated tumors (Fig. 4b).

**Fig. 5:**
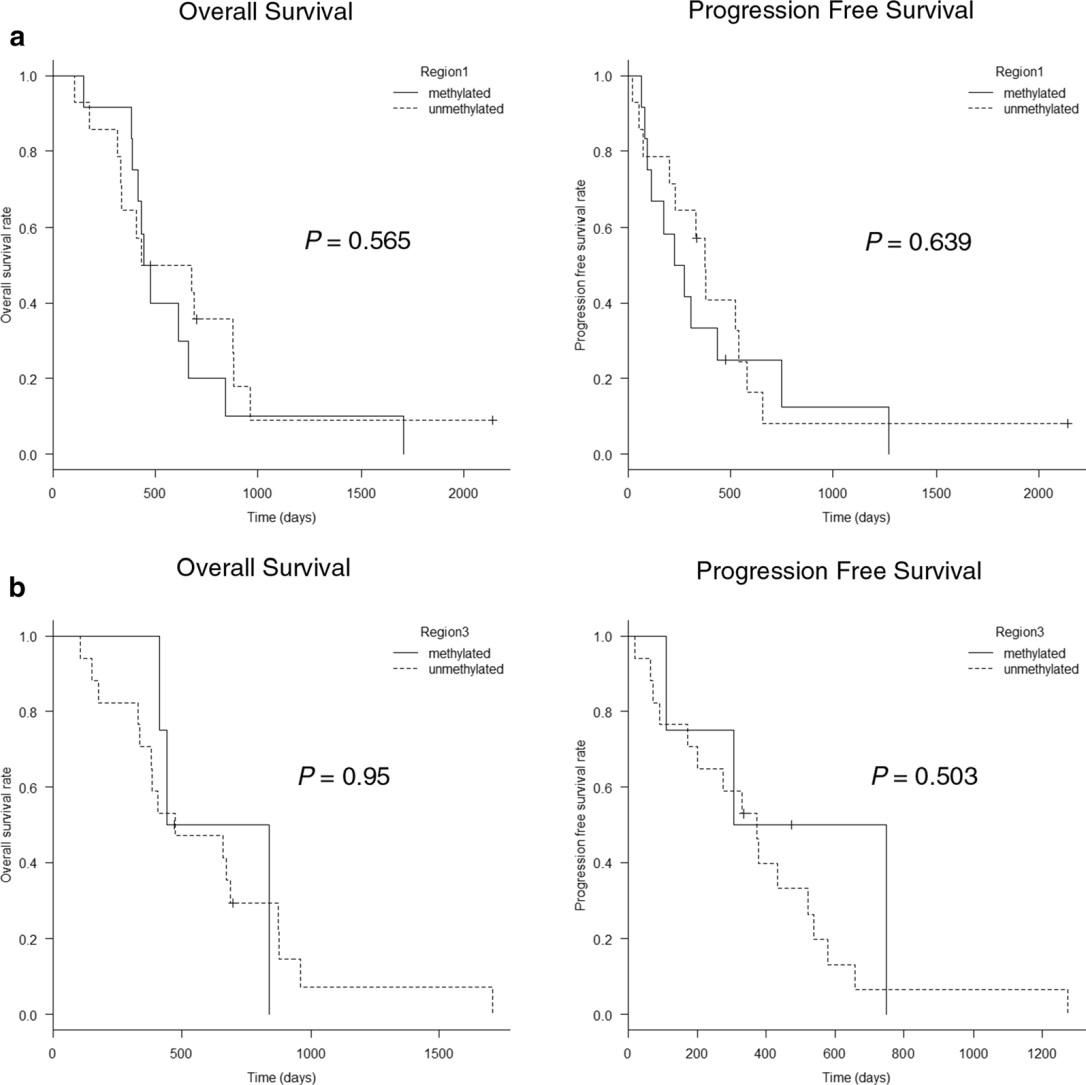
Kaplan–Meier curve based on *MGMT* promoter methylation status determined using FFPE sections. Log-rank test results showed no significant difference between groups with methylated and unmethylated *MGMT* for regions 1 and 3 (**a**, **b**)

Next, HPLC analysis was performed using formalin-fixed paraffin-embedded (FFPE) tumor samples from 28 GBM patients. *MGMT* methylation status could be determined for 26 of the 28 tumors (92.9%) in region 1, 8 of the 28 tumors (28.6%) in region 2, and 21 of the 28 tumors (75%) in region 3. Unadjusted Kaplan–Meier plots of OS and PFS are shown in Fig. 4. Log-rank test results revealed no significant differences between the groups with methylated and unmethylated *MGMT* for regions 1 and 3 (Fig. 5a, b). *MGMT* promoter methylation was observed in 46.2% of region 1 and 19.0% of region 3.

**Fig. 6:**
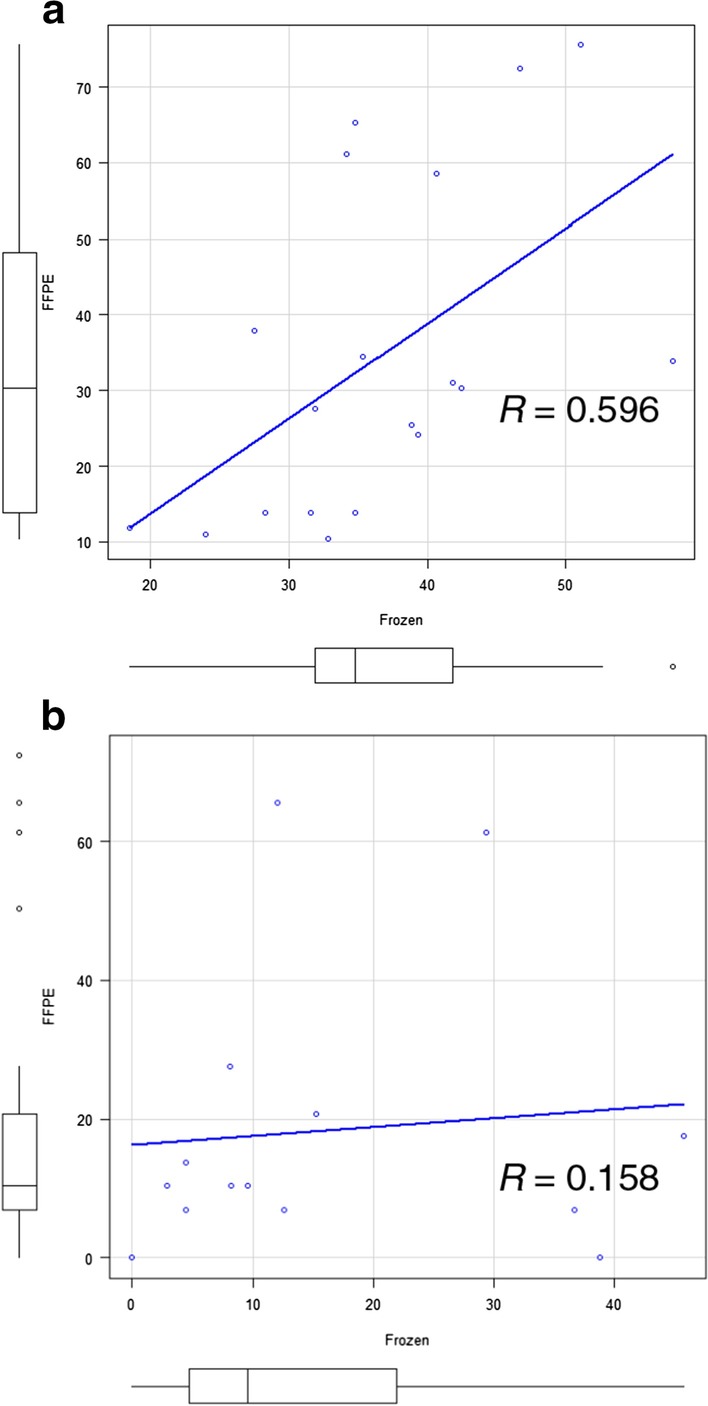
Correlation of results obtained using frozen samples versus FFPE. Agreement between frozen and FFPE samples in terms of *MGMT* methylation in region 1 based on Spearman’s rank correlation (**a**). There was no agreement for region 3 (**b**)

We compared the results obtained using frozen and FFPE samples to determine the usefulness of the HPLC method for analysis of both sample types. Spearman’s rank correlation analysis indicated a moderate correlation between frozen and FFPE samples in region 1 (Fig. 6a). There was no correlation in region 3 (Fig. 6b). We could not determine the methylation status of region 2 for the majority of FFPE samples.

We also analyzed clinical factors affecting OS (Table 2). Univariable analysis revealed that age < 70 years (*p* = 0.0003) and *MGMT* methylation in region 2 (*p* = 0.0026) were associated with better OS. A preoperative Karnofsky Performance Status score of < 70 affected OS positively (*p* = 0.06).

**Table 2:** Association of clinical and genetic factors with overall survival

Factor	Group	n	Median survival (d)	*p* value
Age (y)	> 70	20	660 (413–876)	0.00030
	≤ 70	8	333 (105–432)	
Sex	Male	17	475 (385–735)	0.39
	Female	11	412 (175–841)	
Pre-KPS	> 70	16	409 (314–611)	0.060
	≤ 70	12	689 (432–881)	
EOR	Gross total resection	25	475 (406–689)	0.50
	Partial resection	3	432 (105-NA)	
BCNU wafer	No	19	660 (381–876)	0.11
	Yes	9	441 (149–611)	
Bevacizumab	No	20	453.5 (335–674)	0.75
	Yes	8	565 (314–881)	
Region 1	Methylated	13	674 (406–876)	0.27
	Unmethylated	8	422 (105–881)	
Region 2	Methylated	13	841 (413–961)	0.0026
	Unmethylated	8	363 (105–660)	
Region 3	Methylated	3	876 (406-NA)	0.65
	Unmethylated	18	521.5 (331–735)	

*Pre-KPS* Preoperative Karnofsky Performance Status

## Discussion

The present study examined the diagnostic power of *MGMT* methylation analysis using ion-exchange HPLC for GBM. The patients included in the study were all newly diagnosed with IDH–wild-type GBM and treated with a standard regimen at a single center. The results of the present study provide evidence of a new *MGMT* promoter region (region 2) that encompasses DMR 1 and can be prognostic in IDH–wild-type GBM.

One of the most commonly used methods for *MGMT* methylation analysis is methylation-specific PCR (MSP) [16], which has been adopted in many clinical trials [6, 8, 9]. A major limitation of MSP, however, is that it detects only fully methylated promoters and cannot detect heterogeneous patterns of methylation [17]. Pyrosequencing (PSQ) can overcome this disadvantage, and several studies reported that PSQ is the optimal technique [12, 18]. Disadvantages of PSQ include a requirement for expensive equipment and capacity to analyze only a limited number of sequences up to 100 bp in length [19]. Therefore, analysis of *MGMT* methylation using PSQ could cover only a relatively short section of heterogeneous CpG island methylation. Even using PSQ, a relationship between methylation and expression of *MGMT* could be confirmed for only 85% of patients [14].

Other epigenetic mechanisms could explain the factors affecting *MGMT* transcription. Recently, single CpG methylation of heat shock protein B2 (HSPB2) was reported to have predictive value as a novel epigenetic signature for IDH–wild-type GBM [20]. Therefore, inconsistencies in *MGMT* methylation testing results can be explained not only by differences in methodology, but also by other epigenetic factors. As such, we cannot exclude borderline or gray-zone results in *MGMT* methylation tests. However, in clinical settings, more accurate and cost-effective methods are needed.

In this study, we used three novel bisulfite PCR primers to determine average *MGMT* methylation. We tested methylation in 21 to 38 CpGs of longer length compared to those analyzed in previous studies [10, 12, 21, 22]. In most previous studies, *MGMT* methylation was analyzed in DMR 2, which is located in the gene body, because the GC-rich MGMT promoter region limits bisulfite primer design. Our HPLC method can analyze relatively long PCR products compared to PSQ, and thus, we were able to verify three regions including the promoter. In tumors exhibiting heterogeneous methylation, it could be better to determine the average methylation rate of long CpG islands than the methylation of a specific CpG site. Our HPLC method enables accurate calculation of the average methylation rate.

Furthermore, for region 2, which encompasses DMR 1, the cutoff value that best correlated with prognostic outcome was 8.84% methylation of *MGMT*. The cutoff value of 8.84% and the ratio of methylated to unmethylated *MGMT* (62:38) were consistent with the results of a previous study [23]. However, methylation analysis in region 3, which encompasses DMR 2 and was thought to be the most reliable region, was not correlated with prognosis. The increase in the number of CpGs with increasing length of the PCR product was suggested as the cause of this result. Although previous studies assessed methylation levels of less than 10 CpGs in DMR 2, our HPLC method allowed analysis of the average methylation rate for 38 CpGs in region 3. The higher number of CpGs could be related to differential methylation levels. We demonstrated that methylation in regions 1 and 2 is associated with prognosis, and ours is thus the first report showing the usefulness of analyzing such a relatively long CpG island in the *MGMT* promoter area.

The methylation status could not be determined using our HPLC method for several samples from FFPE sections, particularly for region 2. Although we analyzed the correlation of methylation status between frozen and FFPE samples, FFPE samples were found to be unsuitable, as they contain degraded DNA, which results in even shorter fragment lengths after digestion. Furthermore, the extraction efficiency and DNA quality are poor for older FFPE samples, which precludes high-quality molecular analyses and could affect the results of *MGMT* methylation analyses [24]. The present results thus suggest that frozen samples are most appropriate for HPLC analysis of *MGMT* methylation.

Table 3 shows a comparison of established protocols with the present study protocols in MGMT methylation analysis. Simple, robust, and cost-effective methods are required for commercial *MGMT* methylation testing. Our HPLC method costs only a few dollars per test, and the procedure only involves transferring the PCR product to a vial before HPLC analysis, which takes 10 min. The equipment is small, and the HPLC column can be used thousands of times. Our new method is thus useful and provides excellent quality and cost-effectiveness in comparison with conventional methods.

**Table 3:** Comparison of established protocols with the present study protocols in *MGMT* methylation analysis

Assay	Target CpGs	Strengths	Weaknesses	References
MSP (qMSP)	DMR2 (CpG 71–86, 76–87)	Simple Low cost	Unable to analyze heterogeneous methylation	[12, 17]
PSQ	DMR2 (CpG 74–78)	High accuracy	High cost Limitation of Amplicon length	[12, 23]
MS-HRM	DMR2 (CpG 72–83, 72–89, 84–89)	Capable of analyzing heterogeneous methylation	Low accuracy Limitation of Amplicon length	[10, 12, 22]
HPLC	500 bp upstream of TSS (CpG 1–20) DMR1 (CpG 22–59) DMR2 (CpG 72–98)	Simple Low cost Capable of analyzing long amplicons	Unstable measurement of paraffin-embedded specimen	Present study

CpG numbers on *MGMT* CpG islands were defined by Harris et al. [16]

*DMR* differentially methylated region, *MSP* methylation-specific polymerase chain reaction, *PSQ* pyrosequencing,

*qMSP* quantitative MSP, *MS-HRM* methylation-sensitive high-resolution melting analysis, *TSS* transcription start site

One limitation of this study was the small number of patients. As such, we could not perform a multivariate analysis of *MGMT* methylation. However, the patients included in this study had undergone standardized therapy at a single center, and the significant differences observed among the small number of patients underscore the accuracy of our HPLC method and the strong prognostic impact of *MGMT* methylation testing in GBM patients.

## Conclusions

In neuro-oncology, molecular biomarker–based diagnosis and decision making are routine in clinical settings. *MGMT* promoter methylation is one of the most important markers in patients with GBM. The combination of our HPLC method and original primers enabled the stratification of GBM patients into two groups based on *MGMT* promoter methylation status. Further analyses involving a larger number of patients are needed, however, to confirm our HPLC method as the gold standard for *MGMT* methylation testing.

## Methods

### Patients and tissue specimens

The study included patients newly diagnosed with GBM and treated at the Department of Neurosurgery, University of Yamanashi, between 2007 and 2018. Patients had histologically proven GBM based upon WHO 2016 criteria (International Agency for Research on Cancer [IARC], 4th edition) [25]. IDH1 mutation status was verified by immunohistochemical staining with monoclonal anti-R132H-IDH1 antibody (1:4000; Dianova, Hamburg, Germany). The expression of IDH1 mutant protein was determined semiquantitatively by assessing the proportion of positively stained tumor cells. Cases in which > 10% of cells were positive were defined as involving IDH1 mutation.

HPLC analysis was performed on 28 newly diagnosed GBM patients who underwent maximum resection, followed by radiotherapy plus concomitant and adjuvant TMZ using the Stupp regimen [26]. IDH1 R132H mutation status was verified by immunohistochemical staining and was found to be negative in these patients.

Tissue specimens were acquired after surgical removal, immediately frozen, and stored at − 80 °C until analysis. For FFPE tissue specimens, removed tumors were fixed in 10% buffered formalin and embedded in paraffin. A total of 28 FFPE GBM tumor samples were obtained, and frozen sections were prepared for 23 tumors. All tumor samples were obtained at the time of surgery after patients provided informed consent. This study was approved by the University of Yamanashi (authorization no. 1800) and performed in accordance with all relevant guidelines and regulations.

### DNA extraction

DNA was extracted from fresh frozen tissue samples using a DNeasy Blood & Tissue kit (QIAGEN) or from FFPE tissues using a QIAamp DNA FFPE Tissue kit (QIAGEN) according to the manufacturer’s instructions.

### Bisulfite modification and PCR

A total of 200 ng of genomic DNA was modified with sodium bisulfite using an EZ DNA Methylation-Lightning kit (Zymo Research). The DNA was amplified by PCR using TaKaRa EpiTaq™ HS (Takara Bio). PCR primers for bisulfite PCR were designed using MethPrimer (https://www.urogene.org/methprimer/) (Table 1).

### HPLC

DNA methylation analysis using HPLC was performed as described previously [13]. Briefly, HPLC was performed on an LC-20A system (Shimadzu Corp., Kyoto, Japan) equipped with a stainless steel column (150 × 4.6 mm I.D.) filled with anion-exchange packing material, an oven, an auto-injector, a photodiode array detector, a degassing module, and a data analysis system. Eluent A was 25 mmol/L MES-NaOH buffer (pH 6.0), and eluent B was the same buffer containing 2 mol/L guanidine sulfate. Bisulfited PCR products were separated on a gradient of 30–50% eluent B for 10 min at a flow rate of 1.0 mL/min. The separated PCR products were detected at 260 nm. HPLC analysis was performed at a column temperature of 70 °C. Methylation rate was calculated based on a calibration curve using synthetic DNA fragments of three regions corresponding to 0% (TPG) and 100% (CpG) methylation (Fig. 2a–c).

### Bisulfite NGS

Amplicon libraries were generated using an Ion Plus Fragment Library kit (ThermoFisher Scientific, MA, USA) as described previously [27]. Sequencing was performed using an Ion PGM Hi-Q View Sequencing kit (ThermoFisher Scientific) with 850 flows on an Ion 318 Chip kit v2 (ThermoFisher Scientific), according to the manufacturer’s protocol. After sequencing, single processing and base calling were performed using Torrent Suite 5.12.1 (ThermoFisher Scientific). Methylation analysis was performed using Methylation Analysis_Amplicon plug-in v2.1 (ThermoFisher Scientific).

### Statistical analysis

All statistical analyses were performed using EZR, version 2.3-1 (Saitama Medical Centre, Jichi Medical University, Saitama, Japan), a graphical user interface for R (R Foundation for Statistical Computing, Vienna, Austria) [28]. Average methylation level was calculated for both HPLC and NGS analyses. *MGMT* methylation levels in the same tumor samples were correlated using Spearman rank correlation. The threshold enabling detection of a favorable outcome, defined as longer OS, was identified by ROC curve analysis. PFS and OS were calculated from the time of surgery until recurrence, death, or last follow-up. PFS and OS were estimated using the Kaplan–Meier method, and data were compared using the two-sided log-rank test. Univariate Cox regression analysis was applied to assess the prognostic and predictive values of methylation status of the *MGMT* promoter. A *p* value < 0.05 was considered statistically significant.

## Data Availability

The datasets used and analyzed during the present study are available from the corresponding author on reasonable request.

## Abbreviations

DMR: Differentially methylated region
DNA: Deoxyribonucleic acid
GBM: Glioblastoma
HPLC: High-performance liquid chromatography
HSPB2: Heat shock protein B2
IDH: Isocitrate dehydrogenase
MGMT: O^6^-methylguanine-DNA methyltransferase
MSP: Methylation-specific polymerase chain reaction
NGS: Next-generation sequencing
OS: Overall survival
PFS: Progression-free survival
PSQ: Pyrosequencing
TMZ: Temozolomide
